# JAK1抑制剂治疗复发/难治性大颗粒淋巴细胞白血病疗效及安全性评估

**DOI:** 10.3760/cma.j.cn121090-20260306-00120

**Published:** 2026-06

**Authors:** 林珠 田, 乐乐 张, 若难 李, 伟望 李, 文燕 王, 婧余 赵, 建平 李, 能 聂, 星鑫 李, 静 张, 均 施

**Affiliations:** 1 北京协和医学院，中国医学科学院血液病医院（中国医学科学院血液学研究所），血液与健康全国重点实验室，国家血液系统疾病临床医学研究中心，细胞生态海河实验室，天津 300020 State Key Laboratory of Experimental Hematology, National Clinical Research Center for Blood Diseases, Haihe Laboratory of Cell Ecosystem, Institute of Hematology & Blood Diseases Hospital, Chinese Academy of Medical Sciences & Peking Union Medical College, Tianjin 300020, China; 2 天津医学健康研究院，天津 301600 Tianjin Institutes of Health Science, Tianjin 301600, China

**Keywords:** 白血病，大颗粒淋巴细胞, JAK1抑制剂, 戈利昔替尼, 血液学缓解, Leukemia, large granular lymphocytic, JAK1 inhibitor, Golidocitinib, Hematologic response

## Abstract

**目的:**

评估JAK1抑制剂戈利昔替尼治疗复发/难治性大颗粒淋巴细胞白血病（LGLL）的疗效与安全性。

**方法:**

回顾性分析2025年1月至12月在中国医学科学院血液病医院接受戈利昔替尼单药口服治疗的16例复发/难治性LGLL患者的临床资料（T细胞LGLL 13例，自然杀伤细胞LGLL 3例），起始剂量为0.15 g/d，评估其疗效及安全性。

**结果:**

患者总体中位年龄为58（26～76）岁，基线中位HGB为58.5（43.0～101.0）g/L，中位中性粒细胞绝对计数为1.04（0.02～4.18）×10⁹/L，中位淋巴细胞绝对计数为1.59（0.38～7.20）×10⁹/L，中位大颗粒淋巴细胞计数为0.60（0.06～3.63）×10⁹/L；6例继发纯红细胞再生障碍。中位随访时间为4.0（0.8～11.1）个月，总体血液学缓解率为87.5％（14/16），中位达血液学缓解时间为2.9（1.4～7.6）周，其中血液学完全缓解率为43.8％（7/16）。药物治疗相关不良反应发生率为75.0％（12/16），其中5例患者发生3级不良事件，包括3级粒细胞减少2例、3级血小板减少2例、3级天冬氨酸氨基转移酶升高1例，其余均为1～2级。

**结论:**

戈利昔替尼单药在复发/难治性LGLL患者中具有一定疗效，起效较快，短期安全性可控。

## Abstract (en)

**Objective:**

To evaluate the efficacy and safety of monotherapy with the JAK1 inhibitor golidocitinib in patients with relapsed/refractory large granular lymphocytic leukemia（LGLL）.

**Methods:**

We retrospectively analyzed 16 patients with relapsed/refractory LGLL who received oral golidocitinib monotherapy at the Institute of Hematology & Blood Diseases Hospital, Chinese Academy of Medical Sciences, between January and December 2025（13 with T-LGLL and 3 with NK-LGLL）. The starting dose was 0.15 g once daily, and efficacy and safety were evaluated.

**Results:**

The median age was 58 years（range, 26–76 years）. At baseline, the median HGB level was 58.5 g/L（range, 43.0–101.0）, the median absolute neutrophil count was 1.04×10⁹/L（range, 0.02–4.18）, the median absolute lymphocyte count was 1.59×10⁹/L（range, 0.38–7.20）, and the median absolute large granular lymphocyte count was 0.60×10⁹/L（range, 0.06–3.63）. Six patients had secondary pure red cell aplasia. With a median follow-up of 4.0（0.8–11.1）months, the overall hematologic response rate was 87.5％（14/16）, with a median time to hematologic response of 2.9（1.4–7.6）weeks. The complete hematologic response rate was 43.8％（7/16）. The incidence of drug-associated adverse events（DAE）was 75.0％（12/16）. Five patients experienced grade 3 adverse events, including grade 3 neutropenia in 2 cases, grade 3 thrombocytopenia in 2 cases, and grade 3 elevation of aspartate aminotransferase in 1 case. All remaining DAEs were grade 1–2.

**Conclusion:**

Golidocitinib monotherapy showed activity in patients with relapsed/refractory LGLL, with a rapid onset of response and a manageable short-term safety profile.

大颗粒淋巴细胞白血病（LGLL）是一类来源于细胞毒性T细胞或自然杀伤（NK）细胞的慢性淋巴增殖性疾病[1]–[2]。环磷酰胺、环孢素A及甲氨蝶呤作为一线治疗选择，有效率为50％～70％[3]–[6]，复发/难治性LGLL患者缺乏安全、有效的靶向治疗策略。针对LGLL病理机制的研究证实JAK-STAT通路在大颗粒淋巴细胞（LGL）的增殖、毒性功能方面有关键调控作用[2],[7]–[9]，靶向该通路可能是潜在治疗策略。戈利昔替尼作为口服的选择性JAK1抑制剂，在复发/难治性外周T细胞淋巴瘤的Ⅱ期研究中缓解率达44.3％，显示出良好的安全性[10]，可能为复发/难治性LGLL患者提供新的治疗选择。本研究回顾性分析了戈利昔替尼在16例复发/难治性LGLL患者中的疗效与安全性，为该类患者的治疗选择提供临床依据。

## 病例与方法

1. 病例：本研究回顾性分析2025年1月至2025年12月在中国医学科学院血液病医院接受戈利昔替尼单药治疗的16例复发/难治性LGLL患者的临床资料。其中3例为NK细胞LGLL（NK-LGLL），13例为T细胞LGLL（T-LGLL）。所有患者均符合LGLL诊断标准[11]–[12]。本研究采用连续入组方式，纳入患者需符合以下标准：明确诊断为T/NK-LGLL；既往一线治疗失败；存在明确治疗指征（如进行性中性粒细胞减少、贫血或相关症状等）；一般状况良好，重要脏器功能基本正常，能够耐受相关治疗。排除标准包括：存在活动性感染；合并严重心、肝、肾等重要脏器功能不全；存在其他明确治疗禁忌证或研究者评估不适合接受相关治疗者。本研究经中国医学科学院血液病医院伦理委员会批准（批件号：IIT2025043-EC-1）。

2. 治疗方案：所有患者均接受戈利昔替尼单药0.15 g/d，口服。分别于治疗4周、8周、12周及末次随访进行疗效评价。6例患者于随访中减量，其中5例达到血液学部分缓解（HPR）后减量，1例达到血液学完全缓解（HCR）后减量。例1达HPR后减量为0.15 g，每3天服用2次，后进一步调整为0.15 g，每2天服用1次；达到HCR后1.2个月停药，停药后短期复发，恢复0.15 g，每2天服用1次，治疗3.6周再次达HCR。例3因肝功能异常暂停用药15 d；停药期间维持HPR；恢复治疗后1.9个月达到HCR，随后逐步减量至0.15 g，每3天服用1次。例6达HPR 4.2个月后减量至0.15 g，每2天服用1次；减量后0.4个月达HCR，随后再次减量至0.15 g，每3天服用1次。例7因肝功能异常停药，停药前疗效达HPR。例9、11及15分别于达到HPR后减量至0.15 g，每2天服用1次，其中例9及例15后续达到HCR，例11维持HPR。例16达到HPR后曾短暂停药10 d，停药期间疗效维持HPR，后恢复原剂量治疗并持续至末次随访。

3. 疗效与安全性评价：血液学疗效判定标准参照文献[13]–[15]：HCR：血细胞计数恢复至同年龄段正常水平，且LGL计数降至正常范围；HPR：外周血细胞计数较基线明显改善但尚未完全恢复正常，中性粒细胞绝对计数>0.5×10⁹/L但<1.5×10⁹/L，或输血需求较治疗前减少但仍需输血支持；治疗无效（NR）：患者治疗8周或末次随访时仍未见上述治疗反应。血液学缓解包括HCR与HPR。本研究随访期间出现的不良反应均按照常见不良事件评价标准（CTCAE）5.0版进行分级。

4. 随访：通过查阅门诊/住院病历、电话、微信等方式随访。随访截止日期为2025年12月20日。主要随访指标为血常规、肝功能、肾功能及LGL表型等。无进展生存（PFS）期定义为自开始本研究治疗至疾病进展、复发、死亡或末次随访的时间。

5. 统计学处理：采用SPSS（29.0.1.0版本）进行统计分析。计量资料以*M*（*Q*_1_，*Q*_3_）或*M*（范围）表示；计数资料以例数（％）表示。

## 结果

1. 患者一般特征：如表1所示，16例复发/难治性LGLL患者中，男性11例（68.8％）；中位年龄58（26～76）岁。中位病程32.5（12.0～189.0）个月。所有患者既往均接受过一线免疫抑制治疗（环孢素A、甲氨蝶呤或环磷酰胺），13例为复发患者，3例既往治疗无效。16例（100％）患者均有贫血表现，6例（37.5％）继发纯红细胞再生障碍。12例（75.0％）粒细胞减少，其中3例（18.8％）表现为粒细胞缺乏。2例（12.5％）血小板减少。中位CD3^+^T细胞计数1 044（83～5 419）个/µl，中位CD3^+^CD4^+^T细胞计数243（43～705）个/µl、中位CD3^+^CD8^+^T细胞计数808（43～5 130）个/µl。所有患者均存在克隆性LGL，9例（56％）LGL计数>0.5×10^9^/L。中位淋巴细胞绝对计数为1.59（0.38～7.20）×10^9^/L，中位LGL计数为0.60（0.06～3.63）×10^9^/L。15例患者接受高通量测序，其中10例（66.7％）合并STAT3基因突变。

**表1:** 16例接受戈利昔替尼治疗的复发/难治性大颗粒淋巴细胞白血病患者的基线特征

基线特征	统计值
性别［例（％）］	
男性	11（68.8）
女性	5（31.3）
年龄［岁，*M*（范围）］	58（26～76）
疾病类型［例（％）］	
T-LGLL	13（81.3）
NK-LGLL	3（18.8）
病程［月，*M*（范围）］	32.5（12.0～189.0）
既往用药种数［种，*M*（范围）］	4（1～7）
HGB［g/L，*M*（范围）］	58.5（43.0～101.0）
ANC［×10⁹/L，*M*（范围）］	1.04（0.02～4.18）
PLT［×10⁹/L，*M*（范围）］	208.5（59.0～360.0）
ALC［×10⁹/L，*M*（范围）］	1.59（0.38～7.20）
Ret［×10^9^/L，*M*（范围）］（12例）	46.5（3.0～184.5）
CD3⁺T细胞［％，*M*（范围）］（15例）	95.0（2.0～99.0）
CD3⁺CD8⁺T细胞［％，*M*（范围）］（15例）	59.0（1.0～93.0）
效应CD8⁺T细胞［％，*M*（范围）］（13例）	52.78（1.69～84.94）
LGL计数［×10⁹/L，*M*（范围）］	0.60（0.06～3.63）
LGL计数>0.5×10⁹/L［例（％）］	9（56.3）
STAT3基因突变［例（％）］（15例）	10（66.7）

**注** LGLL：大颗粒淋巴细胞白血病；T-LGLL：T细胞LGLL；NK-LGLL：自然杀伤细胞LGLL；HGB：血红蛋白；ANC：中性粒细胞绝对计数；PLT：血小板计数；ALC：淋巴细胞绝对计数；Ret：网织红细胞计数；LGL：大颗粒淋巴细胞。缺失数据按实际可获得例数统计

2. 疗效评估：中位随访时间为4.0（0.8～11.1）个月。14例患者接受戈利昔替尼后达血液学缓解，总体血液学缓解率为87.5％，中位达血液学缓解时间为2.9（1.4～7.6）周，7例患者达HCR（43.8％），中位达HCR时间为17.4（6.9～24.9）周（图1）。随访期间HGB达到的最佳水平同基线对比中位上升值为58（41，79）g/L。STAT3突变组（10例）的血液学缓解率为90.0％（9/10），无突变组（5例）为80.0％（4/5）。NK-LGLL组（3例）血液学缓解率为100％（3/3），且均达HCR，T-LGLL组（13例）血液学缓解率为84.6％，HCR率为30.8％。两组中位达血液学缓解时间分别为1.9（1.4～2.6）周与3.4（1.7～7.6）周。截至末次随访，对患者进行初步的PFS分析，由于随访期间进展事件较少，中位PFS期尚未达到；平均PFS期为8.24（95％ *CI*：6.82～9.66）个月。

**图1:**
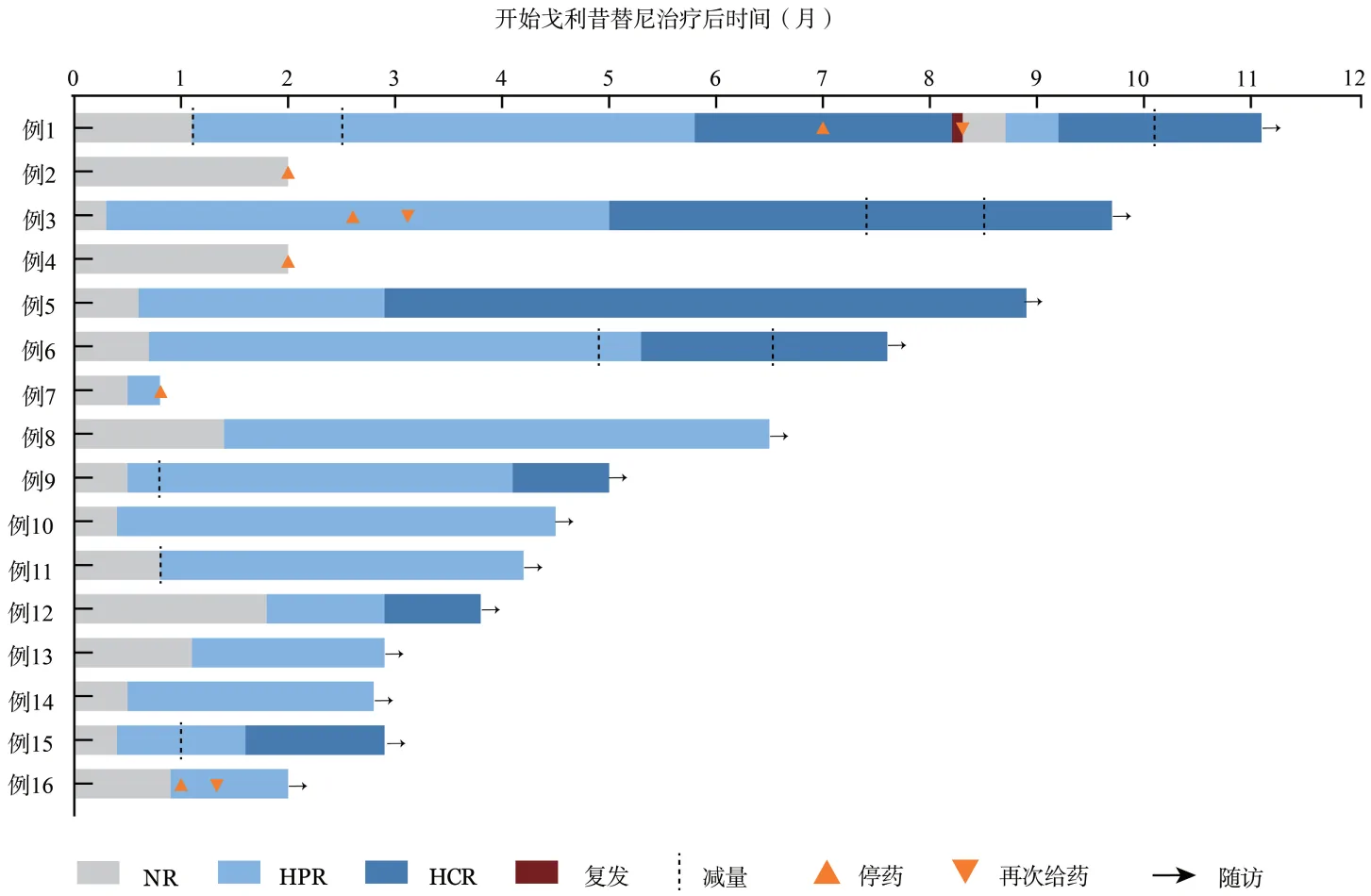
戈利昔替尼治疗16例复发/难治性大颗粒淋巴细胞白血病患者疗效 **注** NR：治疗无效；HPR：血液学部分缓解；HCR：血液学完全缓解

随访过程中共9例患者减量或停药。例2及例4因治疗无效停药，例7因肝损停药，停药前疗效达HPR。6例患者达到血液学缓解后减量（例1、3、6、9、11、15），减量后疗效均维持稳定：4例在减量维持治疗期间由HPR进一步缓解达HCR，1例维持HPR，1例维持HCR。其中例1曾因疗效稳定停药1个月后复发，重启治疗3.6周后再次达HCR。

3. 安全性评估：患者在接受戈利昔替尼治疗期间常规给予抗感染预防，包括抗病毒以及预防卡氏肺孢子菌肺炎。随访期间共12例（75.0％）患者出现药物相关不良反应，以血液学不良反应为主。4例（25.0％）出现粒细胞减少，包括2例3级粒细胞减少、1例2级粒细胞减少及1例1级粒细胞减少；7例（43.8％）出现血小板减少，包括2例3级血小板减少、1例2级血小板减少及4例1级血小板减少。非血液学不良反应多为1～2级，肝功能异常较常见（37.5％），包括2例2级丙氨酸氨基转移酶（ALT）升高、2例1级ALT升高；1例3级天冬氨酸氨基转移酶（AST）升高及5例1级AST升高。3例（18.8％）患者出现1级肌酐升高。感染相关事件以轻中度为主，随访期间共4例患者发生6例次感染事件：上呼吸道感染2例次（12.5％），包括1例次1级和1例次2级；尿道感染1例次（2级，6.3％）、唾液腺感染1例次（2级，6.3％）、咽炎1例次（2级，6.3％）以及带状疱疹1例次（1级，6.3％），经对症及抗感染治疗后缓解。其中2例患者的4例次感染事件均发生于治疗开始后1个月内，余2例患者分别于治疗后4个月发生1例次1级带状疱疹、治疗后6个月发生1例次1级上呼吸道感染。

其他不良反应包括关节疼痛1例（1级，6.3％）、痤疮1例（1级，6.3％）、咳嗽1例（1级，6.3％）及口腔溃疡1例（2级，6.3％）。

## 讨论

戈利昔替尼能够阻断多种依赖JAK1介导的细胞因子信号通路，抑制异常细胞毒性T细胞活化与克隆扩增[16]。戈利昔替尼在复发/难治性外周T细胞淋巴瘤中已表现出良好的抗肿瘤活性与可控的安全性[10]，提示其对成熟克隆性T细胞增殖性疾病具有治疗价值。已有个案报道提示戈利昔替尼治疗NK-LGLL有效[17]。2025年公布的一项前瞻性研究中评估了戈利昔替尼用于复发/难治LGLL患者的疗效与安全性[18]，结果显示总体缓解率（ORR）为92.3％，HCR率为61.15％。本研究LGLL患者的早期血液学缓解率较高，起效较快，且短期安全性总体可控。本队列HCR率低于既往前瞻性研究，可能与中位随访时间较短、部分患者仍处于缓解过程中以及入组人群差异相关。

一线免疫抑制治疗的T-LGLL患者，可以采用环孢素A、甲氨蝶呤、环磷酰胺的替换策略，既往研究提示上述方案有效率30％～85％，但存在起效慢、需长期维持、不良反应大及复发率高等限制因素[3]–[4],[13],[19]。本研究结果显示戈利昔替尼起效更快（中位起效时间3周），可在短期内显著改善贫血及中性粒细胞减少，可为免疫抑制治疗无效或不耐受患者提供新的治疗选择。既往研究显示，在复发/难治性T-LGLL患者中，口服环磷酰胺治疗的ORR为68％，其中完全缓解率为27％，部分缓解率为41％；治疗4个月时ORR为27％，达到最大疗效的中位时间为6个月[20]，提示传统免疫抑制治疗虽具有一定疗效，但总体起效相对较慢。本研究戈利昔替尼治疗复发/难治性LGLL的总体血液学缓解率为87.5％，HCR率为43.8％，中位达缓解时间为2.9周，起效快，能够在较短时间内改善血细胞减少。安全性方面，低剂量环磷酰胺治疗总体耐受性较好，主要为轻度不良反应；本研究中戈利昔替尼相关不良反应总体亦可控，多数不良反应为1～2级，以血液学不良反应和肝功能异常较为常见。需要指出的是，两项研究在疾病类型、样本量、随访时间及疗效评价标准等方面存在差异，本研究样本量较小、随访时间总体较短，对不良事件的分析主要采用描述性统计，相关结果主要提示戈利昔替尼在短期起效方面可能具有一定优势。

本研究中10例患者存在STAT3突变，既往研究报道的比例大致相同（50％～70％）[2]。但并未观察到STAT3突变与疗效差异的显著统计学关联，可能与样本量过小有关。5例无STAT3突变的患者中仍有4例达血液学缓解，提示其疗效机制可能较为复杂，但本研究未对相关分子机制进行深入探讨，尚缺乏直接证据支持。本研究中3例患者为NK-LGLL，血液学缓解率高，受限于样本量小，未在NK-LGLL与T-LGLL患者中发现戈利昔替尼的疗效差异，后续可通过大样本量队列进一步评估。

综上所述，戈利昔替尼在复发/难治性LGLL中展现出良好的短期疗效和安全性，为该类患者提供了新的治疗思路。本研究为单中心回顾性研究，样本量有限，随访时间较短，部分患者尚未达到最佳疗效，上述因素可能对疗效评估造成一定影响，后续应通过前瞻性研究进一步明确其长期疗效、维持方案及停药后复发模式，优化复发/难治性LGLL的标准化管理路径。
